# Expression of concern: Structural, morphological, electrical, and dielectric properties of Na_2_Cu_5_(Si_2_O_7_)_2_ for ASSIBs

**DOI:** 10.1039/d4ra90155j

**Published:** 2024-12-23

**Authors:** Mohamed Ben Bechir, Mehdi Akermi

**Affiliations:** a Laboratory of Spectroscopic and Optical Characterization of Materials (LaSCOM), Faculty of Sciences, University of Sfax 3000 Sfax Tunisia mohamedbenbechir@hotmail.com; b Department Physics, College of Sciences, Jazan University P. O. Box 114 Jazan Kingdom of Saudi Arabia makermi@jazanu.edu.sa; c Laboratory of Interfaces and Advanced Materials, Faculty of Science, Boulevard of the Environment, University of Monastir Monastir Tunisia

## Abstract

Expression of concern for ‘Structural, morphological, electrical, and dielectric properties of Na_2_Cu_5_(Si_2_O_7_)_2_ for ASSIBs’ by Mohamed Ben Bechir *et al.*, *RSC Adv.*, 2024, **14**, 9228–9242, https://doi.org/10.1039/D4RA01454E.

*RSC Advances* is publishing this expression of concern in order to alert readers about concerns that have been raised regarding the BVS analysis and the NPD measurement. An expression of concern will continue to be associated with the article until a conclusive outcome is reached.

Laura Fisher

17th December 2024

Executive Editor, *RSC Advances*

